# Examining learning coherence in group decision-making: triads vs. tetrads

**DOI:** 10.1038/s41598-021-00089-w

**Published:** 2021-10-14

**Authors:** Tsutomu Harada

**Affiliations:** Graduate School of Business Administration, Kobe University, Kobe, Japan

**Keywords:** Cognitive neuroscience, Human behaviour

## Abstract

This study examined whether three heads are better than four in terms of performance and learning properties in group decision-making. It was predicted that learning incoherence took place in tetrads because the majority rule could not be applied when two subgroups emerged. As a result, tetrads underperformed triads. To examine this hypothesis, we adopted a reinforcement learning framework using simple Q-learning and estimated learning parameters. Overall, the results were consistent with the hypothesis. Further, this study is one of a few attempts to apply a computational approach to learning behavior in small groups. This approach enables the identification of underlying learning parameters in group decision-making.

## Introduction

Division of labor and specialization have significantly increased in modern society, and most of the tasks involved in these processes are carried out by groups^1,2^. Management of groups plays a critical role in achieving greater performance, that in turn hinges on understanding underlying group dynamics. Lewin^3^ devised the term “group dynamics” to refer to the way groups and individuals act and react to changing circumstances. In related literature, group performance has been found to be related to a combination of personality traits^4^, member ability^4,5^, team familiarity, team roles^6^ or leadership styles^7,8^, identity, conformity, psychological safety, and cohesiveness^9–11^.

Although these psychological and sociological factors indeed account for group performance, little attention has been paid to empirically measuring and characterizing the learning properties in group decision-making. Thus, this study does so, aiming to estimate relevant learning parameters by taking a computational approach to group decision-making.

Group decision-making has some advantages over individual decision-making. The former induces more employee involvement and satisfaction^12^, leading to higher performance^13^. However, a number of related studies on group decision-making have supported the proposition that groups rarely outperform their best members^14,15^. Nevertheless, the majority of related literature on group decision-making, under the assumption that group members cooperate and share information voluntarily^15–17^, has shown that groups outperform individuals in decision-making, enabling knowledge transfer from group to individual contexts^18,19^, more accurate information recall^20^, better negotiation outcomes^21^, more creative ideas^22^, and more accuracy^23,24^.

The single detection approach highlights the performance problems that can occur with individuals vs. dyads, the effects of larger group size on performance remain to be examined. This study examined whether three heads perform better than four or one. The comparison of groups of three and four and individuals highlights new, interesting issues in group decision-making that do not arise with groups of two vs. one, that is, even-sized groups vs. odd-sized groups^14,25^. Small groups are likely to break into two coalitions. If a group has an even number of members, the two subgroups are equal in size. In this case, since the majority rule cannot be applied, subgroup dynamics might lead to deadlock^26–29^. In contrast, if a small group has an odd number of members, a minority and majority subgroup emerge, and the majority influence provides a clear direction and group cohesion^14,25,30,31^. Thus, it is predicted that odd-sized small groups have higher cohesion and consistent decision-making, leading to superior performance to even-sized ones.

This hypothesis could be reformulated in terms of learning coherence and incoherence. That is, triads (more generally, odd-sized groups) perform better than tetrads (more generally, even-sized groups) because the former maintains learning coherence due to the majority rule, whereas the latter suffers from learning incoherence due to conflict among group members. To formalize learning coherence in triads and learning incoherence in tetrads, assume two learning strategies exist, Sh and Sl, and the ratio of the Sh in the population is p. The strategies Sh and Sl generate expected rewards of Rh and Rl where Rh > Rl. In addition, a randomized strategy between Sh and Sl could exist, that underperforms Sh and Sl due to its learning incoherence. The expected rewards for this randomized strategy are Rw, where Rw < Rl < Rh. In the tetrads, when two subgroups of two members have different learning strategies, conflicts arise, leading to a situation in which both strategies are randomly adopted. The probability of adopting these strategies is $6{p}^{2}{\left(1-p\right)}^{2}$. In contrast, the triads never encounter this situation because the majority, who decides the preferred strategy, always exists. Thus, learning coherence could be achieved in triads. The difference between expected rewards for the tetrads and triads are $-3{p}^{2}{\left(1-p\right)}^{2}\left[Rh+Rl-2Rw\right]<0$, indicating that tetrads underperform triads.

The purpose of this study was to examine the hypothesis that learning coherence emerges in individuals and triads and learning incoherence occurs in tetrads by estimating and comparing learning parameters.

## Methods

### Participants

A total of 343 healthy undergraduate students at Kobe University (103 women, age range = 19–25 years, SD = 1.21) participated in the study for course credit. All experimental protocols in this study were approved by the Ethics Committee, Graduate School of Business Administration, Kobe University, and the study was carried out in accordance with the relevant guidelines and regulations. All participants signed an informed consent form before the experiment.

### Experiments

In test 1, participants undertook cognitive tasks (two-armed bandit [TAB] problems) individually. In test 2, they formed groups of three and performed as groups. In test 3, they formed groups of four and performed the same cognitive tasks as groups. There were seven rounds of tests. To control for learning effects, three tests were randomly assigned to either groups or individuals in each round, that is, some groups were triads, the other groups were tetrads, and the remaining were individuals. All tests were performed with the PsytoolKit^32,33^, and when participants performed the TAB tasks as a group, they communicated with each other via a breakout session in Zoom to decide the choices in the TAB.

All participants undertook test 1, and most of the participants took part in tests 2 and 3. In each of the triad and tetrad groups, at least one member participated in both tests 2 and 3. Because all group members in the triads did not participate in test 3, and all group members in the tetrads did not participate in test 2, 8 triad groups and 4 tetrad groups were dropped from the sample. As a result, the total number of groups of triads and tetrads examined in this study were 100 and 104, respectively.

### Q-learning model

In this study, a simple Q-learning reinforcement learning algorithm^34^ was adopted to account for asymmetric learning rates (learning biases). Participants played a TAB problem, in which they chose either a right or left box on the screen. After the selection, the participants were awarded either 10 or 0 points, and they were instructed to try to achieve the highest score over a series of 100 choices. One of the boxes had a higher probability of being worth 1 point (70%), and the corresponding probability of the other box was set at 30%. However, we switched these probabilities twice over 100 choices. For example, the right and left boxes had a respective 70% and 30% probability of being worth 1 point for the first 30 choices, and from the 31st to the 70th choice, the probabilities switched such that the probability of earning 1 point for the right and left boxes became 30% and 70%, respectively. Then, for the last 30 choices, the probabilities of the right and left boxes returned to the initial respective levels of 70% and 30%. Thus, in each round of tests 1 and 2, these changes in probability took place three times over 100 choices. Moreover, the probabilities were randomized for every round of tests 1 and 2 so that even in the same test, the probability for each round differed. Therefore, participants could not transfer learning obtained in one round of the test to other rounds.

In the Q-learning framework, a decision-maker is assumed to calculate the action value for each choice (i.e., the right and left boxes). The action value of option *i* at trial *t* is denoted by ${Q}_{i}\left(t\right)$, calculated as follows:

1 $$ \begin{array}{*{20}c} {{\text{Q}}_{i} \left( {t + 1} \right) = \left\{ {\begin{array}{*{20}c} {{\text{ Q}}_{i} \left( t \right) + \alpha^{ + } \delta \left( t \right) + \phi if \delta \left( t \right) \ge 0, } \\ {{\text{ Q}}_{i} \left( t \right) + \alpha^{ - } \delta \left( t \right) + \phi if \delta \left( t \right) < 0,} \\ \end{array} } \right.} \\ \end{array} $$

with

2 $$ \begin{array}{*{20}c} {\delta \left( t \right) = R_{i} \left( t \right) - {\text{Q}}_{i} \left( t \right), } \\ \end{array} $$

where ${\mathrm{R}}_{i}\left(t\right)$ is the reward associated with option $i$ at trial $t$, either 10 or 0 points, and $\delta \left(t\right)$ is the reward prediction error. ${\alpha }^{\pm }$ indicates the learning rate so that the learning biases are measured by ${\alpha }^{+}-{\alpha }^{-}$. If this is positive (negative), positivity (negativity) biases exist. $\phi$ is added in Eq. 1 as the choice trace to account for autocorrelation of choice, which could affect learning biases^35^.

As one of the characteristics of learning, this study compared positivity biases. The positivity and confirmation biases refer to the tendency to respond to positive news more sensitively than to negative news, and the tendency to respect outcomes consistent with one’s hypothesis^36^. Related studies examined the existence of these biases in individual reinforcement learning, and reported that learning rates tend to be positively biased^37–41^. Katahira^35^ suggested that the autocorrelation of choices itself tends to generate pseudo-positivity biases. Harada^42^ controlled for this autocorrelation by incorporating the effects of past choices into the learning model, and demonstrated that the positivity biases were indeed confirmed in a simple Q-learning model. However, once a more dynamic model was introduced, the positivity biases disappeared. Therefore, learning biases not only depended on the autocorrelation of choices, but also on autocorrelation of learning parameters in the model. While previous studies examined learning biases for individuals, this study investigated the existence of positivity biases in group learning of triads and tetrads. As related studies indicated, it could be inferred that either positivity biases existed or no biases existed for both triads and tetrads. According to our hypothesis, we speculated that learning coherence in triads lead to positivity biases because individual learning was reported to generate positivity biases in related studies while tetrads generated no biases due to learning incoherence.

If the decision-maker $\mathrm{j }\left(\mathrm{i}\ne \mathrm{j}\right)$ does not choose the option, its action value does not change, remains to be changed:

4 $$\begin{array}{c}{Q}_{j}\left(t+1\right)={Q}_{j}\left(t\right).\end{array}$$

Given these action values of the two options, the decision-maker determines one of the two options according to the softmax decision rule:

5 $$\begin{array}{c}P\left(a\left(t\right)=i\right)=\frac{exp\left(\beta {Q}_{i}\left(t\right)\right)}{\sum_{j=1}^{2}exp\left(\beta {Q}_{j}\left(t\right)\right)},\end{array}$$

where $P\left(a\left(t\right)=i\right)$ is the probability of choosing the action $a\left(t\right)=i$ at trial $t$. The parameter $\upbeta$ is the inverse temperature, that measures the relative strength of exploitation vs. exploration (exploitation/exploration ratio). Exploitation is related to optimization under current contexts, implying the choice of the option with the highest action value ${Q}_{i}\left(t\right)$. Exploration, on the other hand, refers to the digression from optimization so that one of the options without the highest action value is selected. If $\upbeta$ is high, the probability of choosing the option with the highest action value increases, leading to exploitation. In contrast, if $\upbeta$ is low, the probability of choosing the option without the highest action value increases. Thus, $\upbeta$ measures the exploitation/exploration ratio.

### Estimation method

The parameters specified in Eqs. (1)–(5) were estimated by optimizing the maximum a posteriori objective function:

6 $$\begin{array}{c}\widehat{\theta }=argmax \; p\left({D}_{s}|{\theta }_{s}\right)\mathrm{p}\left({\theta }_{s}\right),\end{array}$$

where $p\left({D}_{s}|{\theta }_{s}\right)$ is the likelihood of data ${D}_{s}$ for a subject $\mathrm{s}$ conditional on parameters ${\theta }_{s}=\left\{{{\alpha }^{\pm }}^{S}{, \phi }^{S},{\beta }^{S}\right\}$. $\mathrm{p}\left({\theta }_{s}\right)$ is the prior probability of ${\theta }_{s}$. Note that $\mathrm{\alpha }$ should be bounded between 0 and 1, and $\upbeta$ take non-negative values. Therefore, the corresponding priors were assumed to follow beta distributions for ${\alpha }^{\pm }$ with shape parameters of 2 and 2, and gamma distributions for $\upbeta$ with a shape parameter of 2 and a scale parameter of 3. In addition, ${\phi }^{S}$ is assumed to follow standard normal distribution with mean 0 and variance 1.

## Results

This study investigated underlying learning mechanisms of triads and tetrads from two perspectives: (1) group differences and (2) within-group effects. The descriptive statistics for relevant variables are reported in Table 1. Since the data rejected either the homogeneity of variance by the Bartlett test or the normality by the Shapiro–Wilk test in the statistical tests of the differences of relevant data across and within groups, the Kruskal–Wallis test was applied in the subsequent analyses without referring to the results of either the Bartlett or the Shapiro–Wilk tests, due to space limitation.

**Table 1:** Descriptive statistics.

	Individuals		Triads		Tetrads	
	Mean	SD	Mean	SD	Mean	SD
**Performance**	49.35	6.3	51.19	7.69	49.01	5.11
Max	–	–	53.68	4.02	54.44	3.47
Min	–	–	45.34	4.24	41.86	9.93
Average	–	–	49.68	3.29	48.7	3.69
**α**^**+**^**-α**^**-**^	0.08	0.34	0.1	0.35	− 0.12	0.15
Max	–	–	0.39	0.2	0.4	0.18
Min	–	–	− 0.21	0.27	− 0.27	0.22
Average	–	–	0.09	0.2	0.07	0.18
**β**	4.36	3.01	5.57	3.56	2.33	1
Max	–	–	6.67	2.62	7.82	2.59
Min	–	–	2.01	1.46	1.79	1.38
Average	–	–	4.2	1.62	4.52	1.6

### Group differences

#### Performance

**Figure 1:**
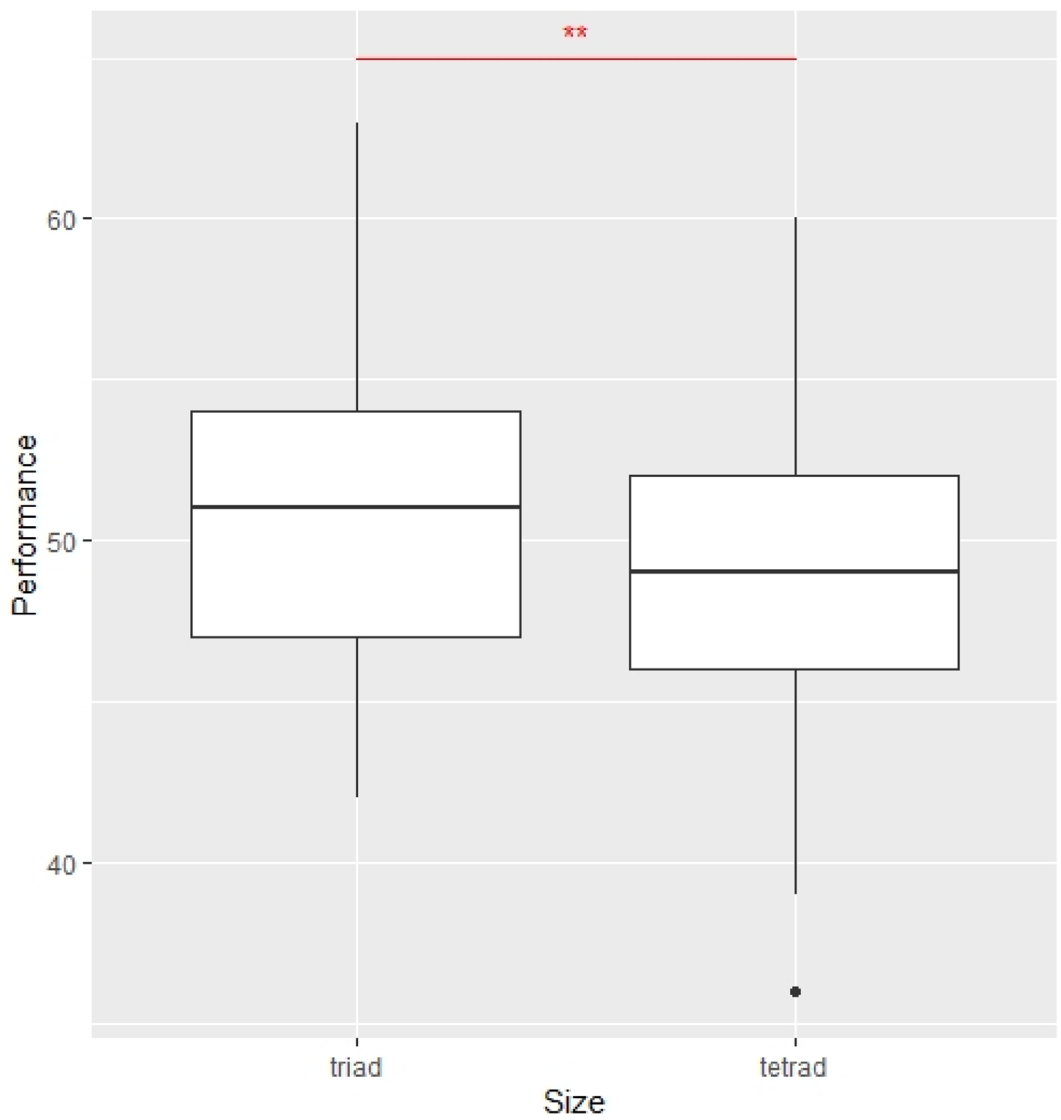
Comparison of average performance of triads and tetrads. Error bars represent standard errors of means. The Kruskal–Wallis test test was applied. **p < .05.

First, the performance difference between triads and tetrads was examined. The result suggested that a performance difference existed between triads and tetrads and triads outperformed tetrads (${\chi }^{2}$=4.12, p = 0.04). Thus, we could identify that triads generated slightly higher performance than tetrads (see Fig. 1).

#### Inverse temperature

**Figure 2:**
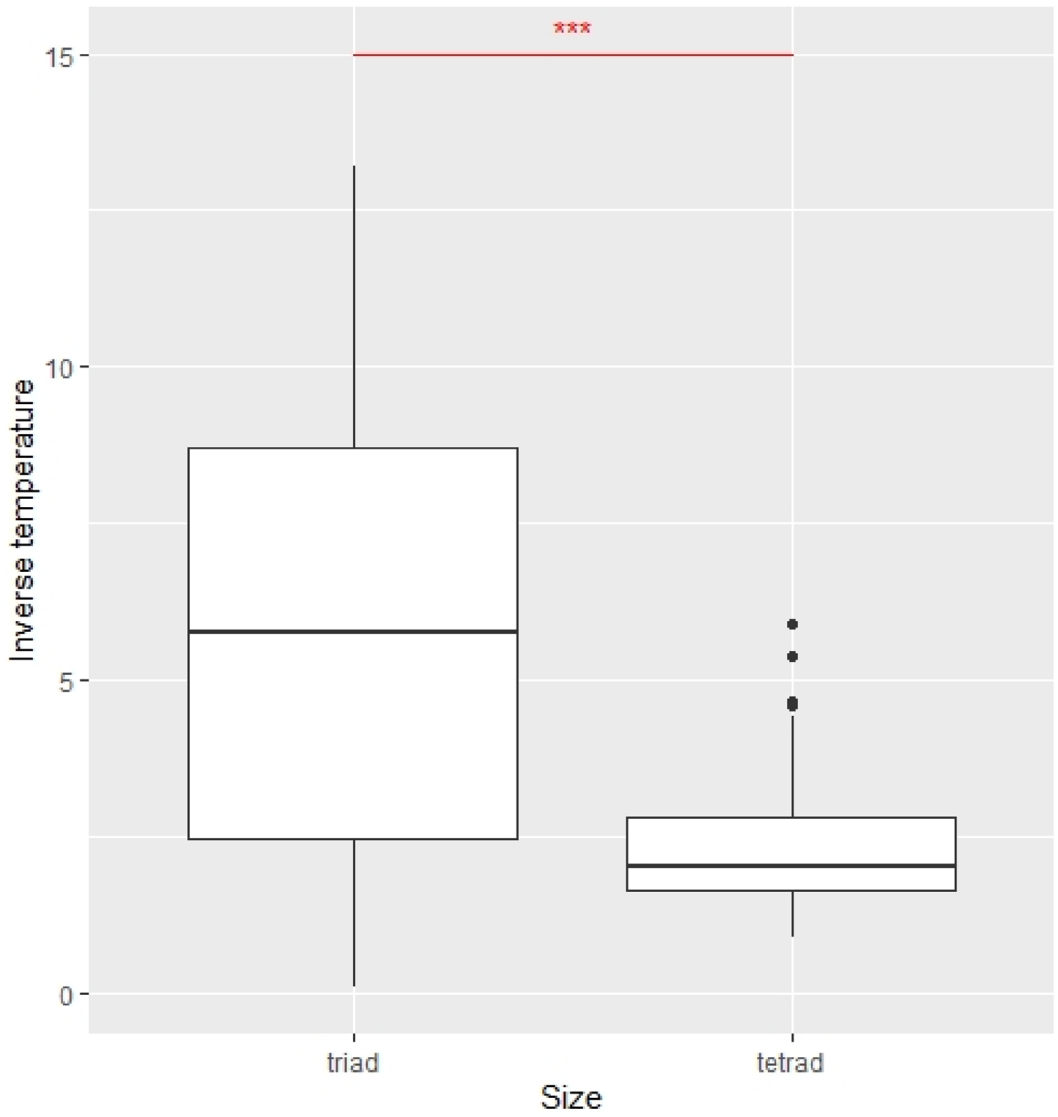
Comparison of average inverse temperatures ($\upbeta )$ of triads and tetrads. The Kruskal–Wallis test was applied. ***p < .01.

As the first characteristic of learning, the magnitude of the inverse temperature between triads and tetrads was compared. Inverse temperature measured the degree of exploitation vis-à-vis exploration. Exploitation adopts the optimal choices, given existing information, whereas exploration makes random choices. Inverse temperature was significantly higher for triads than for tetrads (${\chi }^{2}$=42.88, p = 5.8.e−11) (see Fig. 2). It follows that triads were more likely to make random choices, regardless of past records. It could be inferred that this result was generated due to the fact that the majority rule was harder to apply in tetrads than in triads. This implied learning coherence in triads and incoherence in tetrads.

#### Positivity biases

**Figure 3:**
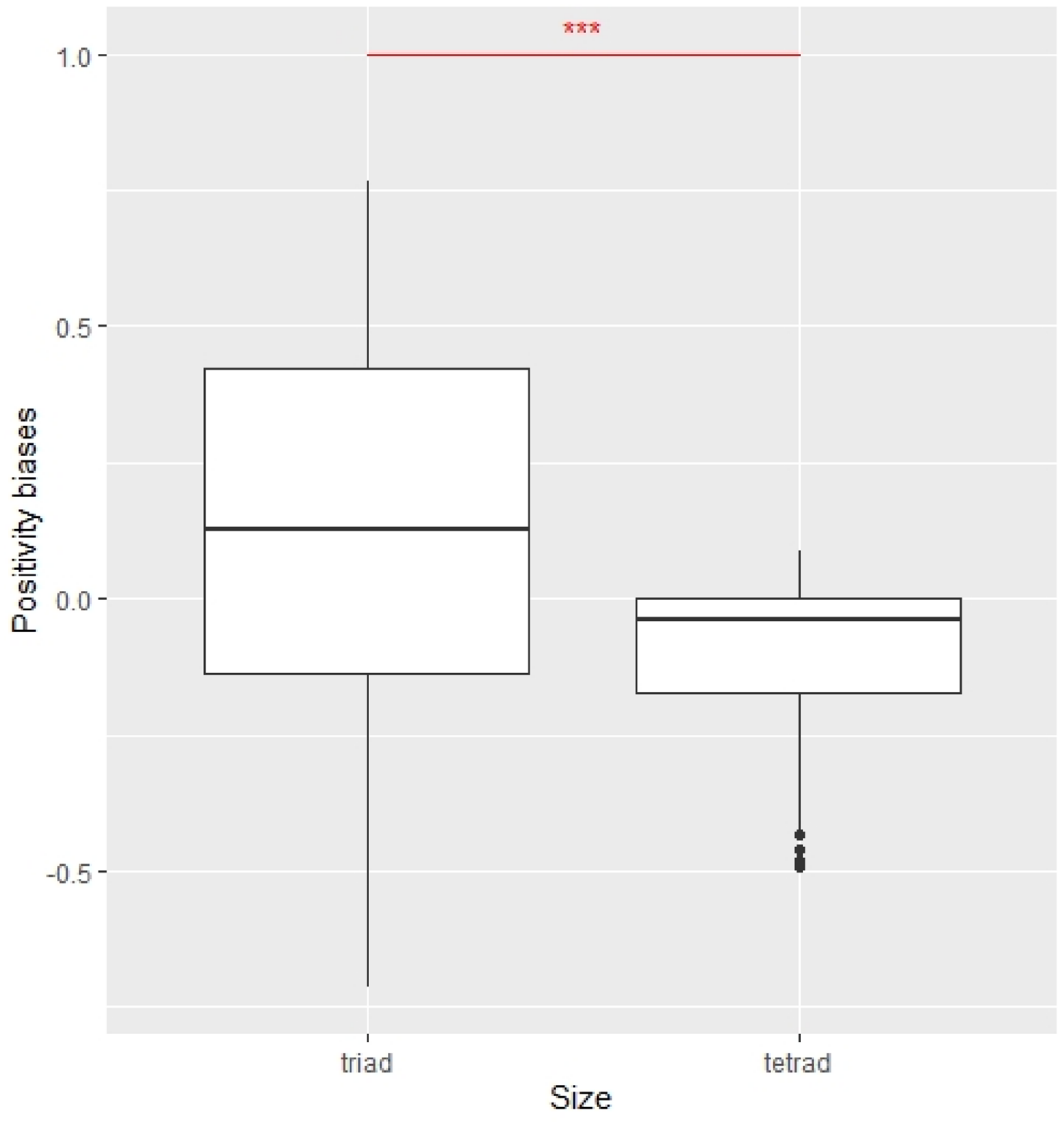
Comparison of average positivity biases (${\alpha }^{+}-{\alpha }^{-}$) of triads and tetrads. The Kruskal–Wallis test was applied. ***p < .01.

As the second characteristic of learning, this study compared positivity biases. While previous studies examined learning biases for individuals, this study investigated the existence of positivity biases in group learning of triads and tetrads. As related studies indicated, it could be inferred that either positivity biases existed or no biases existed for both triads and tetrads. For triads, the positivity biases were supported (${\chi }^{2}$=13.39, p = 2.5e−04). However, for tetrads, we confirmed negativity biases (${\chi }^{2}$=24.05, p = 9.4e−07). This study also investigated learning biases for individuals, revealing that positivity biases existed (${\chi }^{2}$=22.08, p = 2.6e−06). Thus, while individuals and triads confirmed positivity biases, tetrads generated negativity biases (see Fig. 3). According to related studies, this result suggested learning coherence for triads and learning incoherence for tetrads.

### Within-group effects

As the within-group effects, the maximum, minimum, and the average of group members’ individual performances and learning parameters were compared with the corresponding group variables.

#### Performance

In triads, group performance outperformed the minimum of individual performances of group members (${\chi }^{2}$=45.7, p = 1.4e-11), but underperformed its maximum version (${\chi }^{2}$=23.91, p = 1.1e-06). However, group performance and the average of individual performances were not differentiated (${\chi }^{2}$=0.89, p = 0.34). Similarly, in tetrads, group performance outperformed the minimum of individual members (${\chi }^{2}$=47.94, p = 4.4e-12), but underperformed both their maximum (${\chi }^{2}$=59.46, p = 1.3e-14). Group performance and its average version were not differentiated (${\chi }^{2}$=0.03, p = 0.87).

#### Inverse temperature

In triads, inverse temperature was greater than its minimum (${\chi }^{2}$=58.41, p = 2.1e−14) and average (${\chi }^{2}$=7.12, p = 0.01) of individual group members, but was weakly smaller than its maximum version (${\chi }^{2}$=3.50, p = 0.06). In tetrads, group inverse temperature was greater than the minimum of individual members (${\chi }^{2}$=26.68, p = 2.4e-07), but was smaller than both its maximum (${\chi }^{2}$=137.48, p = 2.2e-16) and average versions (${\chi }^{2}$=87.80, p = 2.2e-16).

Thus, group effects in triads were higher in inverse temperature because triads achieved higher group inverse temperature than their average, while those in tetrads were smaller than their average version.

#### Positivity biases

In triads, positivity biases were greater than the minimum of individual performances of group members (${\chi }^{2}$=40.02, p = 2.5e-10), but were smaller than its maximum version (${\chi }^{2}$=26.05, p = 3.3e-07). However, positivity biases and their average version were not differentiated (${\chi }^{2}$=1.03, p = 0.31). In tetrads, negativity biases were greater than all of their minimum (${\chi }^{2}$=34.33, p = 0.4.7e-09), maximum (${\chi }^{2}$=142, p = 2.2e-16), and average (${\chi }^{2}$=46.45, p = 9.4e-12) of individual members.

Thus, group effects in triads were high in generating positivity biases, but those in tetrads were also significant in giving rise to negativity biases.

## Discussion

Overall, our statistical analysis revealed that triads had higher performance, higher inverse temperature, and more positivity biases. Since inverse temperature and positivity biases were indicated to be positively related to performance, these results implied that triads achieved learning coherence, but tetrads experienced learning incoherence. On the one hand, it can be inferred that triads that might break into majority and minority subgroups, enabled the group to achieve consistent and efficient learning over 100 choices, indicated by high performance, inverse temperature and positivity biases. On the other hand, tetrads that might be constrained by two equal subgroups, encountered dispute and confrontation, sometimes leading to deadlock, resulting in lower performance and inconsistent learning behavior, represented as low inverse temperature and high negativity biases. These results were consistent with related studies^14,25–29,31^.

Of course, dispute and confrontation do not necessarily impair group performance. For example, in more creative tasks that require insight and experimentation, the high exploration observed in tetrads might be more efficient than triads subjected to majority influence. However, in the TAB problems, insight and experimentation were not required. Instead, utilizing past information and efficiently guessing an advantageous box played a critical role in achieving higher performance, that in turn hinged on consistency in learning strategies. Thus, while this study confirmed that odd-sized groups (i.e., triads) performed better than even-sized groups (i.e., tetrads) in learning tasks that do not require creativity or insight, tetrads might be superior to triads in creative tasks and insight problem-solving. This could be an interesting research topic in the future.

In contrast to performance, inverse temperature, positivity biases, risk parameters, $\upmu ,$ and $\upnu$, did not account for the difference between triads and tetrads. Note that risk-seeking behavior also has a tendency toward divergence from current learning strategies. In this sense, risk-seeking has some similarity to exploration. However, in our model, exploration corresponded to divergence from the optimal Q value, that already incorporated risk-seeking behavior. Hence, risk-seeking and exploration have subtle differences. That inverse temperature differed between triads and tetrads, implying that the divergence from a consistent learning strategy was reflected in the inverse temperature but not in risk attitudes.

In addition to these results, this paper contributes a novel methodology for the study of small groups. To the best of our knowledge, this is one of the first attempts to take a computational approach to the study of small-group dynamics. Of course, a large body of literature on group dynamics has empirically investigated the properties of the dynamics of small groups. However, most of these studies did not explicitly model the underlying mechanism of group decision-making or estimate parameters that characterize group dynamics. The computational approach proposed in this paper articulates the algorithm of group decision-making and enables the underlying learning parameters to be estimated, allowing for rigorous comparison among small groups in terms of learning parameters such as inverse temperature and risk attitudes. We hope this computational approach sheds new light on group dynamics and group decision-making.

In this respect, it should also be noted that a simple Q-learning model, or reinforcement learning in general, closely correspond to the actual working of neural networks in the brain. The key variables are the actual rewards and reward prediction errors. The Q value is the expected reward, that is updated by feedback from a reward prediction error. This reinforcement learning framework is supported by a number of empirical studies including neural signals in various cortical and subcortical structures that behave as predicted^43–46^. For example, it is now commonly accepted that dopamine neurons in the midbrain of humans and monkeys encode reward prediction errors^46–48^. Thus, the reinforcement learning model class is typically matched by brain activity. Since the simply Q-learning model considered in this paper belongs to this model class, the model matches brain activity, unlike abstract and unrealistic models without an empirical foundation.

One of the managerial implications derived from this study is that group size is crucial to the management of small groups. In particular, when groups undertake learning under uncertainty without the burden of creativity and insight, triads, rather than tetrads, should be selected. However, when tasks require much creativity and insight, tetrads, rather than triads, might be preferred, although this idea was not examined in this study. In broader contexts, odd-sized groups are favored for learning tasks without creativity and even-sized groups for creative problem-solving^25^. This rule is clear and straightforward to implement, but, of course, diversity in knowledge, skill, working experiences, cultural backgrounds, and personalities also account for group performance. However, unless managers have sufficient time to take these factors into account, this simple rule should be implemented.

Finally, we would like to point out the limitations of this study. First, while we confirmed that triads outperformed tetrads, learning coherence of triads and learning incoherence of tetrads were inferred from the results on inverse temperature and positivity biases, rather than derived from a strong theoretical background. In this sense, these learning characteristics were exploratory in our hypothesis. In future studies, more detailed learning mechanisms generating learning coherence and incoherence should be specified and empirically tested. Second, learning tasks (TAB) are fundamental to the results of this study. If different kinds of tasks are assigned, the relative performance of triads and tetrads would differ. In particular, as described above, insight problem-solving or creative tasks might have opposite results regarding the relative performance of triads and tetrads. This constitutes one of our future research challenges.

## Conclusion

This study focused on the relative performance and learning characteristics of triads and tetrads as an extension of Simmel^49^ research on dyads vs. triads to triads vs. tetrads, and also serves as a specific investigation of odd- vs. even-sized group dynamics^14,25^. Generally, our study confirmed that the odd-sized groups performed better than the even-sized groups. Moreover, it was revealed that learning coherence and incoherence were observed in triads and tetrads, respectively. In addition to the confirmation of the theoretical predictions, this study developed a new computational model that enables the estimation of the underlying learning properties of small groups. In related works, Harada^50^ also showed that individuals and triads performed better than dyads due to learning coherence of individuals and triads and the learning incoherence of triads. This study was consistent with this result in that the odd-sized groups (triads) performed better than even-sized groups (tetrads). To the best of our knowledge, this study was one of a few attempts to apply the reinforcement learning framework to group decision making.
